# A Systematic Review of How Cardiopulmonary Bypass Parameters Influence Electroencephalogram Signals

**DOI:** 10.3390/brainsci16040412

**Published:** 2026-04-13

**Authors:** Han Bao, Jiaying Wang, Ziru Cui, Min Zhu, Wenyi Chen, Liwei Zhou, Georg Northoff, Tao Tao, Pengmin Qin

**Affiliations:** 1 Key Laboratory of Brain, Cognition and Education Sciences, Ministry of Education, Center for Studies of Psychological Application, School of Psychology, South China Normal University, Guangzhou 510631, China; han_bao@m.scnu.edu.cn (H.B.); jiaying_wang@m.scnu.edu.cn (J.W.); 2025023645@m.scnu.edu.cn (Z.C.); 2024024023@m.scnu.edu.cn (W.C.); liweizhou9701@gmail.com (L.Z.); 2 Department of Anaesthesia, Zhujiang Hospital of Southern Medical University, Guangzhou 510010, China; 17865523117@163.com; 3 Mind, Brain Imaging and Neuroethics Research Unit, The Royal’s Institute of Mental Health Research, University of Ottawa, Ottawa, ON K1Z 7K4, Canada; georg.northoff@theroyal.ca; 4 Center of Sleep and Brain Medicine, Shenzhen Hospital, Southern Medical University, 13 Xinhu Road, Baoan District, Shenzhen 518000, China; 5 Pazhou Lab, Guangzhou 510330, China

**Keywords:** cardiopulmonary bypass, electroencephalography, neurological outcomes, intraoperative neuromonitoring

## Abstract

**Highlights:**

**What are the main findings?**

- The impact of key physiological (hypothermia, MAP, hemodilution) and technical/pharmacological factors (anesthetic agents and the occurrence of embolization and systemic inflammation) on brain metabolism and perfusion are summarized.
- Key EEG parameters and their clinical significance in CPB are summarized.

**What are the implications of the main findings?**

- Underscore the importance of EEG in perioperative neuromonitoring. Future research should focus on EEG-guided interventions, optimization of CPB management parameters, and validation of novel EEG metrics to improve long-term neurological outcomes in cardiac surgery.
- Specific CPB-related factors produce distinct EEG patterns: frequency slowing, burst suppression (e.g., from hypothermia or certain anesthetics) and epileptiform discharges (e.g., after embolization), which are associated with postoperative neurological complications such as delirium and cognitive decline.

**Abstract:**

Background: Cardiopulmonary bypass (CPB) is an essential technique for cardiac surgery but significantly increases the risk of perioperative neurological complications. Electroencephalography (EEG) enables real-time monitoring of brain function and provides sensitive biomarkers for early detection of cerebral injury. However, a systematic synthesis of how CPB-related physiological, pharmacological, and technical factors influence EEG signals, and how these insights can be integrated into clinical decision-making, is still lacking. Objective: To systematically review the effects of temperature management, mean arterial pressure (MAP), hemodilution, anesthetic agents, embolization, and systemic inflammatory response during CPB on EEG parameters (including frequency bands, Bispectral Index (BIS), quantitative EEG metrics such as burst suppression ratio (BSR), spectral edge frequency (SEF), etc.), and to evaluate the associations between EEG changes and postoperative delirium (POD) and stroke. Methods: Following the PRISMA 2020 guidelines, we searched PubMed, Web of Science, and related databases for original English-language articles published between February 1974 and September 2025. Inclusion criteria: adult patients (≥18 years) undergoing cardiac surgery with CPB and intraoperative EEG monitoring (raw or processed). Exclusion criteria: reviews, case reports, animal studies, pediatric populations, and articles with inaccessible full texts. Two reviewers independently screened the literature and extracted data; a narrative synthesis was performed. Results: Fifty-one studies were included. Main findings: (1) Hypothermia: BIS decreases linearly with temperature (≈1.12 units/°C); electrocerebral silence occurs during deep hypothermic circulatory arrest; EEG recovery dynamics during rewarming predict POD. (2) MAP and cerebral perfusion: The rate of MAP decline (≥0.66 mmHg/s) is a stronger predictor of EEG abnormalities than the absolute MAP value; under fixed pump flow, some patients exhibit coexisting cerebral overperfusion and metabolic suppression. (3) Hemodilution: Maintaining hemoglobin ≥9.4 g/dL prevents EEG slowing; a drop below 9.2 g/dL significantly increases the risk of slowing. A ≥10% decrease in regional cerebral oxygen saturation (rSO_2_) is associated with a 1.5-fold increased risk of burst suppression. (4) Anesthetic agents: Propofol maintains flow-metabolism coupling, and BSR reflects deep anesthesia better than BIS; sevoflurane and isoflurane impair autoregulation and suppress EEG. (5) Embolization and inflammation: EEG epileptiform discharges increase the risk of POD five-fold; a decrease in LIR predicts stroke (AUC 0.771) and POD (AUC 0.779); persistent EEG changes increase the risk of POD 2.65-fold. Conclusions: CPB-related factors affect EEG signals through distinct mechanisms, and specific EEG patterns (slowing, burst suppression, asymmetry, epileptiform discharges) are significantly associated with postoperative neurological complications. Multimodal monitoring (EEG + cerebral oximetry + hemodynamics) with clear intervention thresholds facilitates individualized brain protection. Future interventional studies using real-time EEG feedback are needed to confirm improvements in long-term neurological outcomes.

## 1. Introduction

Cardiopulmonary bypass (CPB) is the mainstay of modern-day heart surgery. It involves the use of an apparatus to temporarily take over the function of the heart and lungs, diverting the circulation from the heart to produce a bloodless surgical field. Numerous designs exist, but most share the same basic principles: oxygenation and removal of carbon dioxide from the blood; circulation of the blood throughout the body, cooling and rewarming the body; and the diversion of blood from the heart [1].

Essential as CPB is to a multitude of complex cardiac procedures, it also has so profound an effect on physiology that the central nervous system (CNS) is rendered especially susceptible to neurological events. Neurological complications of cardiac surgery (from subtle neurocognitive impairment to frank stroke) are common, and the prevalence of neurocognitive dysfunction can be as high as 25% to 80% of patients at hospital discharge, with some still having deficits years postoperatively [1]. Furthermore, the incidence of perioperative stroke in high-risk cardiovascular procedures, such as coronary artery bypass grafting (CABG), ranges from 1.9% to 9.7% [2].

The CPB circuit itself triggers a systemic inflammatory response syndrome (SIRS) with the release of injury inflammatory mediators. The basis for this is blood coming into contact with artificial surfaces, leading to complement activation, leukocyte activation, and endothelial cell dysfunction [3]. In those life-saving cardiac interventions that call for it (CPB), risk to neurological integrity (non-physiological flow, an artificial surface, inflammatory activation) is a simultaneous factor. All of this suggests that however much support is provided to systemic circulation, some sacrifice is necessary due to the potential for neurological compromise, thus indicating the critical need for brain monitoring.

Electroencephalography (EEG) is useful in perioperative neuromonitoring, recording the summated postsynaptic potentials of neural tissue from electrodes on the scalp [4]. It aims to minimize the incidence of intraoperative awareness and the time to waking up, and provides valuable useful information about cerebral perfusion [5]. This electrical activity reflects the state of the brain in real time, making it possible for the clinician to warn of cerebral ischemia, direct the appropriate depth of anesthesia and hypothermia, and detect seizure activity [6].

## 2. Method

### 2.1. Search Strategy and Selection Criteria

The studies summarized in the tables were selected following the Preferred Reporting Items for Systematic Reviews and Meta-Analyses (PRISMA) 2020 guidelines [7]. We performed a systematic search of the PubMed, Web of Science and relevant databases, restricted to peer-reviewed articles published in English between February 1974 and September 2025. The search query was constructed using four categories of keywords, combined using the “AND” operator to ensure high specificity: Category 1: (“EEG” OR “Electroencephalography” OR “Electroencephalographic”). Category 2: (“CPB” OR “Cardiopulmonary bypass”).

### 2.2. Inclusion and Exclusion Criteria

Studies summarized in the tables were screened for eligibility based on the following criteria:

Inclusion Criteria: (1) Publication type: original, peer-reviewed research articles. (2) Language: published in English. (3) Participants: adult patients (≥18 years) undergoing surgery requiring CPB. (4) Modality: must employ intraoperative EEG monitoring, including processed EEG (e.g., Bispectral Index, entropy, SedLine) or raw EEG analysis. (5) Reporting of Findings: must report specific findings related to EEG changes, cerebral oxygenation, hemodynamic parameters, or clinical outcomes (e.g., postoperative delirium, cognitive dysfunction, stroke).

Exclusion Criteria: (1) Reviews, meta-analyses, systematic reviews, commentaries, editorials, conference abstracts, book chapters, or single-case studies. (2) Studies involving non-human subjects (i.e., animal models). (3) Studies with pediatric populations (age < 18 years). (4) Studies without intraoperative EEG monitoring or those not involving CPB. (5) Studies not available in English or with full text inaccessible.

### 2.3. Study Selection and Data Extraction

**Figure 1:**
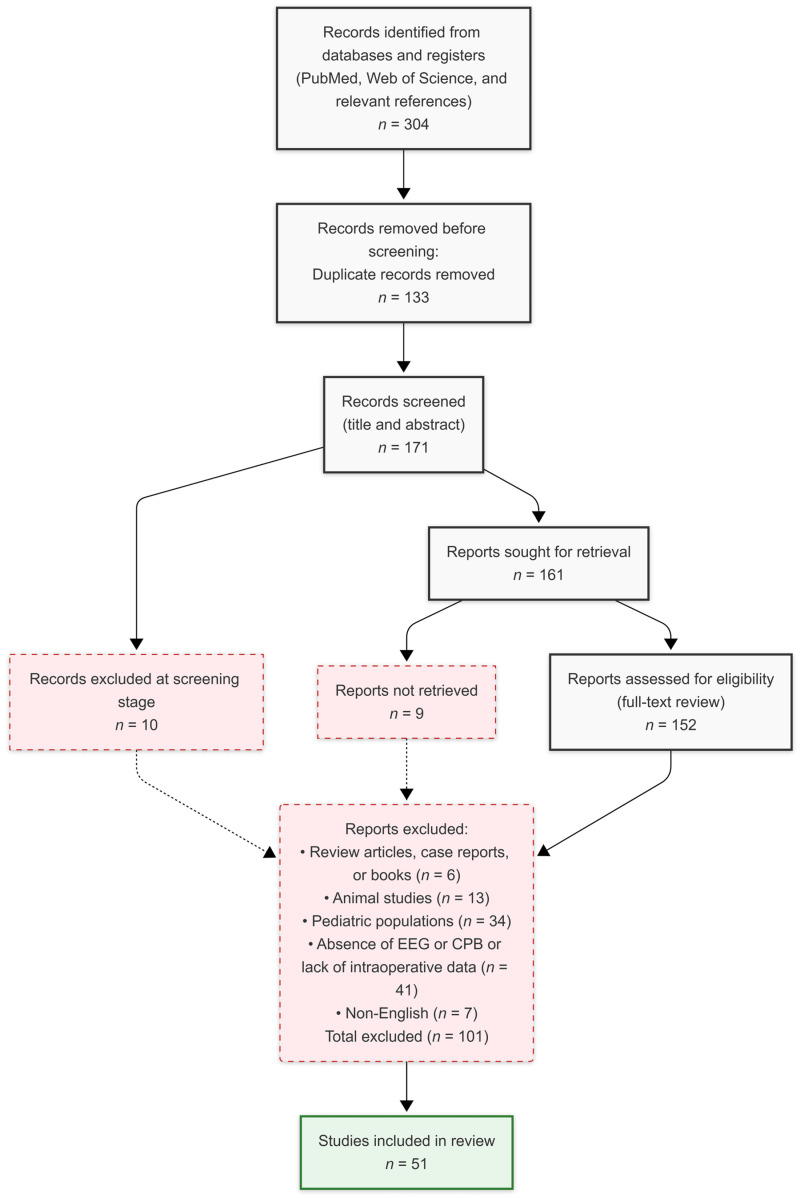
Process for the studies summarized in the tables.

The PRISMA flow diagram details the selection process for the studies summarized in the tables (Figure 1). A total of 155 records were initially identified. After automatic duplicate removal, all remaining titles and abstracts were independently screened for eligibility by two reviewers. Any disagreements were resolved through discussion or consultation with a third reviewer. Full texts of potentially eligible articles were then assessed against the full inclusion/exclusion criteria. Following this process, 51 studies met the inclusion criteria, while 101 were excluded for the following reasons: review articles, case reports, or books (*n* = 6), animal studies (*n* = 13), pediatric populations (*n* = 34), absence of EEG or CPB or lack of intraoperative data (*n* = 41), and non-English (*n* = 7).

Data from all included studies were extracted by one reviewer and subsequently verified by a second reviewer to ensure accuracy. The following information was extracted: bibliographic details (e.g., first author, year of publication); sample characteristics (e.g., sample size, age, gender); surgical and CPB details; EEG monitoring modality and parameters; key findings related to EEG changes, clinical outcomes, and associated factors.

## 3. Fundamentals of EEG Monitoring and Interpretation in Cardiac Surgery

### 3.1. Basic Principles of EEG and Brainwave Frequency Bands (Delta, Theta, Alpha, Beta)

EEG measures the electrical activity of the cerebral cortex, providing a measure of its activity [8,9]. As the patient goes from being alert to unconscious, the normal pattern of small rapid brain waves begins to change to larger ones, typically ranging from 1 to 5 cycles per second. It also increases in amplitude as the depth of anesthesia or brain inactivation deepens [10].

EEG activity is conventionally categorized into distinct frequency bands, each correlating with different brain states and cognitive functions.

Delta Waves (<4 Hz): These denote the lowest frequencies and underlie deep sleep, motivational processes and unconstrained urges [11]. Compared with T1 (30 min after CPB onset), T2 (at CPB end) showed a diffuse increase in delta band. Decrements in EEG power band greater than 10% were detected only for a delta band over 56.5% of electrodes, considering the T2-T1 difference (*p* < 0.05); compared with T0 (before CPB onset), the delta band also increased significantly at T2 (*p* < 0.05) [12].

Theta Waves (4–7 Hz): Theta oscillations have been associated with memory, affect, and stimulus saliency [11]. T2 (at CPB end) vs. T1 (30 min after CPB onset) revealed a diffuse decrease in theta band (*p* < 0.05); T1 vs. T0 (before CPB onset) showed a mild anterior increase in theta power, which was not statistically significant (*p* > 0.05). Hemodilution during cardiopulmonary bypass leads to a decrease in hemoglobin concentration. Although systemic oxygen saturation may remain normal, the actual oxygen supply to the brain is reduced, triggering metabolic suppression in neurons. The alternation between highly hyperpolarized and depolarized states forms the neurophysiological basis for slow waves (delta and theta) [12].

Alpha Waves (7–12 Hz): Alpha activity tends to be inversely correlated with cortical activation, and has been proposed to inhibit unnecessary processing, gate decisions about perceptual awareness, and allow focusing of attention [13,14,15]. Multivariable regression analysis revealed that each 1 dB increase in power within this preoperative frequency band was associated with a 12% reduction in the odds of developing burst suppression (OR = 0.88, 95% CI: 0.79–0.98) and an 11% reduction in burst-suppression duration (IRR = 0.89, 95% CI: 0.84–0.93) [16]. A left frontal alpha band decrease was observed after CPB end (T2), compared with the intra-intervention values (T1) (*p* < 0.05) [12].

Beta Waves (12–30 Hz): This band is studied as part of sensorimotor behavior, in that power decreases during the preparation and the execution of voluntary movements and bursts after their termination [17]. The beta activity supports the activation of attention and alertness [18]. As in alpha waves, intra-operatively decreased power in the beta range was shown to predict susceptibility to burst-suppression during CPB. The underlying neural mechanism is that reduced preoperative (pre-CPB) EEG alpha and beta power reflects impaired integrity of cortical pyramidal neurons and interneurons. This neurophysiological vulnerability makes patients more susceptible to burst suppression at equivalent anesthetic depths [16].

### 3.2. Bispectral Index (BIS) Monitor

The Bispectral Index (BIS) (Aspect Medical, Cambridge, MA, USA) analyzes multiple characteristics of the EEG and condenses these findings into a single, dimensionless numerical value, commonly ranging from 0 (complete cerebral suppression) to 100 (awake state), to objectively detect the presence of cerebral suppression [5].

The value of BIS monitoring during cardiac surgery with CPB has been extensively studied, but its performance varies significantly under different conditions. At the clinical application level, a closed-loop anesthesia control system based on BIS feedback has been proven to be feasible. In cardiac surgery with hypothermic CPB, the system was functional for 96.1% of the time, with BIS maintained within ±10 of the target for 86% of the time (excluding the CPB period), and no patient experienced intraoperative awareness [19]. Furthermore, the availability of BIS information significantly improved anesthesia decision-making: when anesthesiologists were aware of BIS values, the frequency of events decreased by 30% (5.00 vs. 3.49 events per patient, *p* < 0.001), of which 70.5% led to interventions involving anesthetic titration, and the propofol consumption was significantly lower in the BIS-visible group (*p* < 0.0167) [20]. These findings demonstrate the practical value of BIS monitoring in optimizing anesthetic management.

However, the reliability of BIS during the late phase of CPB is controversial. A study found that in the late CPB period, despite no significant change in BIS values (36.0 vs. 35.1, *p* = 0.695), the 95% spectral edge frequency decreased significantly from 14.6 Hz to 10.6 Hz (*p* = 0.0022), approximate entropy decreased from 0.56 to 0.32 (*p* = 0.0076), and bicoherence peaks decreased by 45% and 48% in the delta-theta and alpha bands, respectively. This indicates that the agreement between BIS and other depth-of-anesthesia monitors is limited [21]. Tirén et al. performed a paired comparison of BIS, entropy, and the auditory-evoked potential index with 62% showing good agreement, 33% non-agreement, and 5% showing disagreement, suggesting that these three monitors are not interchangeable [22]. Under normothermic CPB with isoflurane anesthesia, the SNAP™ II (Stryker, Inc., Kalamazoo, MI, USA) values were systematically 28% higher than BIS values [23]. Entropy indices are superior to BIS in terms of artifact resistance; Baulig et al. found that BIS was significantly more affected by frontal EMG interference (r^2^ = 0.62) compared to entropy indices (r^2^ = 0.39–0.45), and the monitoring failure time for BIS accounted for 9.1% of the anesthesia time, whereas for entropy it was only 0.1% (*p* < 0.0001) [24]. Musialowicz et al. further confirmed that although BIS and entropy indices were well correlated (r^2^ = 0.66–0.70), Bland–Altman analysis showed wide limits of agreement (BIS-state entropy (SE): +16 to −11.8; BIS-response entropy (RE): +14.3 to −14.2) and RE was sensitive to surgical noxious stimulation (Cohen’s d = −0.71 after skin incision and −0.94 after sternotomy), suggesting that RE is a useful indicator for assessing patient arousal [25].

In summary, BIS can serve as an effective tool for monitoring the depth of anesthesia and supporting decision-making during cardiac surgery with CPB; however, it has limitations under conditions of hypothermia, during the late CPB period, and in the presence of electrical or mechanical interference. In clinical practice, these limitations should be fully recognized, and when necessary BIS should be combined with other monitoring modalities such as entropy indices to achieve more precise anesthetic management.

### 3.3. Quantitative EEG (qEEG) Parameters

It was demonstrated that 19-lead QEEG analysis during CPB surgery can effectively predict cognitive dysfunction at 2–3 months postoperatively. Among the findings, increased EEG asymmetry and reduced absolute power during surgery were identified as key predictors of long-term cognitive decline. In predicting cognitive dysfunction at 2–3 months postoperatively, the discriminant functions achieved a sensitivity ranging from 69.6% to 87.0% and a specificity ranging from 77.8% to 88.9% [26].

#### 3.3.1. Burst-Suppression Ratio (BSR)

Another EEG pattern of interest is that of burst suppression. It is a characteristic pattern of an inactivated brain and is typically found at deep levels of general anesthesia, during hypothermia, and in pathological conditions including coma and early infantile encephalopathy. The authors propose a unifying biophysical model for burst suppression based on the interaction between neuronal dynamics and brain metabolism. The model integrates cortical neurons with metabolic dynamics mediated by ATP-sensitive potassium (K_ATP) channels. Reduced cerebral metabolism—from anesthetics, hypothermia, or ischemia—lowers ATP production. Neuronal activity consumes ATP, opening K_ATP channels, which hyperpolarizes neurons and halts firing. During suppression, ATP slowly regenerates, closing the channels and allowing activity to resume. This negative feedback loop produces the quasiperiodic burst-suppression pattern [27]. The BSR provides a quantitative measure of this pattern. Assigning 0 to bursting and 1 to suppression permits calculation of the time spent in a suppressed state during a particular time period. A higher BSR indicates less electrical activity of the brain [28].

Under the same anesthetic protocol, individual differences in EEG responses remained significant. Preoperative alpha and beta power (odds ratio (OR) = 0.88, 95% confidence interval (CI): 0.79–0.98; incidence ratio (IRR) = 0.89, 95% CI: 0.84–0.93) indicated that decreased EEG alpha and beta power predicted the later incidence and duration of burst suppression. Although other clinical variables (such as gender, depression, and diabetes) showed differences in group comparisons, they did not reach statistically significance in the regression models, possibly due to the limited sample size [16].

It was found that the incidence of postoperative delirium was 25% in patients who exhibited burst suppression, compared to 6% in those who did not (OR = 3.79; 95% CI: 1.50–9.60, *p* = 0.005). In the multivariable logistic regression model, burst suppression remained significantly associated with delirium after adjusting for other confounders (OR = 4.1, 95% CI: 1.5–13.7, *p* = 0.012). Additionally, age was significantly associated with delirium in univariate analysis (OR = 1.09, 95% CI: 1.02–1.16, *p* = 0.009) and was retained as the only exogenous variable in the multivariable model (OR = 1.07, 95% CI: 0.99–1.15, *p* = 0.090), suggesting a direct effect on delirium. Notably, although age was not included in the final multivariable model for burst suppression, it was significantly associated with burst suppression in univariate analysis (OR = 1.08, 95% CI: 1.03–1.14, *p* = 0.002), and was significantly correlated with physical function scores (r = −0.16) and EEG alpha power (r = −0.33), indicating that age may indirectly influence burst suppression through these factors, thereby affecting the risk of delirium [29].

#### 3.3.2. Spectral Edge Frequency (SEF)

Spectral edge frequency (SEF) is a quantification of the EEG frequency below which a specified percentage (typically, 90% or 95%) of the total power is found [30,31,32]. It is typically visualized on a spectrogram (the distribution of EEG frequencies against time) [33].

SEF has also been exploited as a variable reflecting anesthesia depth [34]; for example, an alpha activity band (8–13 Hz) might reflect surgical anesthesia depth [34] whereas beta activity (>13 Hz) might indicate lighter sedation. Conversely, an SEF value <8 Hz could predict possible anesthetic overdose [35]. It appears that a consistently acceptable SEF interval for adequate general anesthesia has not been established; it is prone to significant variation with respect to anesthetic drugs used and the patient’s age and clinical state [35,36,37]. It was found that in the late period of CPB, the SEF decreased from 14.6 Hz preoperatively to 10.6 Hz (*p* = 0.0022), whereas the BIS showed no significant change (36.0 vs. 35.1, *p* = 0.695). The study demonstrated that SEF is more sensitive than BIS in detecting the slowing of EEG activity during the late phase of CPB, suggesting that SEF may serve as a supplementary indicator for monitoring changes in brain functional status during CPB [21].

Key EEG parameters and their relevance in CPB, as well as clinical strategy, are summarized in Table 1.

## 4. Physiological Factors Influencing EEG Indicators During CPB

### 4.1. Temperature Management: Effects of Hypothermia and Rewarming on Cerebral Metabolism and EEG Activity

Temperature is perhaps the most important physiological variable during CPB, as it exerts a major influence on cerebral physiology and metabolism [1]. Hypothermia has a significant and quantifiable impact on EEG monitoring parameters. Under mild to moderate hypothermic conditions, the BIS decreases by approximately 1.12 units per 1 °C reduction in temperature (*p* < 0.001) [38], with the median BIS in the hypothermic group being significantly lower than that in the normothermic group (41 vs. 49, *p* < 0.0001) [39]. The patient state index (PSI) decreases by 0.84 points per 1 °C during the cooling phase (95% CI: 0.68–0.99, *p* < 0.001), while the burst suppression ratio increases by 2.9% per 1 °C during cooling (95% CI: 2.3–3.4, *p* < 0.001) [40]. Under deep hypothermic circulatory arrest, EEG activity ceases completely when temperatures reach 16–20 °C; however, the exact temperature at which electrocerebral silence (ECS) occurs varies widely among individuals, with esophageal temperatures ranging from 7.2 °C to 23.1 °C (mean 13.6 °C) [41].

Different EEG monitoring parameters exhibit marked differences in their sensitivity and stability during hypothermia. Somatosensory evoked potential (SSEP) latency shows a strong linear correlation with temperature, with the N20 latency changing by 1.56 ms per 1 °C (r^2^ = 0.67, *p* < 0.0001) [42]. Under steady-state hypothermia, N20 amplitude increases by 26% (*p* < 0.001) and latency is prolonged by 17% (*p* < 0.001) [43]. Brainstem auditory evoked potentials (BAEP) are the most stable during hypothermia, remaining recordable at 20–25 °C, whereas cortical SSEP disappears below 25 °C [44]. The Pa latency of middle-latency auditory evoked response (MLR) shows a correlation coefficient of −0.617 with temperature (*p* < 0.01), disappearing below 23 °C but remaining highly sensitive to decreases in blood pressure [45]. In contrast, BIS exhibits wide variability during hypothermia, with a correlation coefficient of only 0.033 with temperature (*p* > 0.05) [46]. When the burst suppression ratio (SR) exceeds 50%, BIS shows a strong linear correlation with SR (BIS = 50.8 − 0.51 × SR, r = −0.987) [47].

EEG changes during the cooling and rewarming phases show clear asymmetry. During cooling, the SEF demonstrates a linear correlation with tympanic membrane temperature (r = 0.76, *p* = 0.26 × 10^−9^), whereas during rewarming, the relationship becomes nonlinear (*p* = 0.011) [48]. Total EEG power increases by 1215 μV^2^ per 1 °C increase in temperature (*p* < 0.0001), and the high-frequency peak frequency increases by 0.39 Hz per 1 °C (*p* < 0.002) [49]. Following deep hypothermic circulatory arrest, the time to BIS recovery is significantly correlated with the duration of circulatory arrest, with the time point at which BIS begins to recover after ECS showing a positive correlation with the duration of arrest [47].

These findings have important implications for clinical anesthesia. The independent effect of hypothermia on BIS (1.12 units per 1 °C) means that clinicians must consider temperature when interpreting depth of anesthesia and should not adjust anesthetic dosing based solely on BIS values obtained during hypothermia [38,50]. Regarding the choice of hypothermic strategy, the moderate hypothermia group (26–31 °C) had a significantly higher area under the curve for regional cerebral oxygen saturation (rSO_2_-AUC) compared with the mild hypothermia group (32–35 °C) (259 vs. 185, *p* = 0.009), as well as a lower incidence of delirium (6.67% vs. 36.36%), suggesting that moderate hypothermia may offer better cerebral protection [51]. By comparing the effects of mild hypothermia (32–34 °C) and moderate hypothermia (28–30 °C) on BIS in patients undergoing CPB, it was found that the moderate hypothermia group had significantly lower BIS values during CPB compared to the mild hypothermia group, particularly at 30 min after the onset of CPB (22.4 ± 7.1 vs. 36.9 ± 11.1, *p* = 0.0007), before aortic cross-clamp release (19.0 ± 11.7 vs. 39.5 ± 17.4, *p* < 0.0001), and after aortic cross-clamp release (14.4 ± 6.5 vs. 43.5 ± 13.0, *p* = 0.0007) [50]. The increase in SSEP amplitude during steady-state hypothermia should not be misinterpreted as neurological improvement [43]. Furthermore, EEG monitoring can help distinguish hypothermia-induced EEG changes from acute ischemic events, as ischemic events typically present with more rapid EEG alterations [49]. Finally, BAEP and short-latency SSEP are more reliable modalities for monitoring brain function during hypothermia [44]. The temperature management data in CPB are summarized in Table 2.

### 4.2. Mean Arterial Pressure (MAP) and Cerebral Perfusion: Impact on Brain Oxygenation and EEG Patterns

The concept of maintaining adequate mean arterial pressure during CPB serves to ensure adequate and continual perfusion of all other vital organs, especially the brain [52]. Despite its importance, there is still debate regarding the optimum MAP intraoperatively and during CPB [1].

The relationship between MAP and cerebral perfusion during CPB is complex and does not follow a simple linear pattern. In one study, MAP was maintained between 50 and 100 mmHg during CPB, and perfusion pressure (the difference between MAP and central venous pressure) was kept above 40 mmHg. The study found that SEF was independent of perfusion pressure during both cooling and rewarming phases, suggesting that EEG changes during hypothermia are primarily temperature-driven rather than pressure-dependent. This finding underscores that EEG can help distinguish hypothermia-induced slowing from acute ischemic events caused by inadequate perfusion [48].

Researchers explored the association between cerebral perfusion and postoperative delirium (POD). The median MAP values were 68 mmHg in the non-delirium group and 74 mmHg in the delirium group, with no statistically significant difference; CPB pump flow was fixed at 2.5 L/min/m^2^ in both groups. They found that patients who developed POD had significantly lower BIS values during CPB (adjusted mean difference: −4.449, 95% CI: −7.978 to −0.925) and significantly higher middle cerebral artery blood flow velocity (MCAV) (10.655 cm/s, 95% CI: 0.491 to 20.819). This suggests a mismatch between cerebral metabolic demand and blood flow, with cerebral overperfusion occurring even when MAP is within conventional targets. Notably, there was no significant difference in cerebral autoregulation indices (Mx and COx) between the two groups, indicating that the observed overperfusion was not attributable to autoregulation failure but rather to a fixed CPB pump flow that exceeded individual metabolic requirements [53].

Researchers highlighted that the rate of hemodynamic change, rather than the absolute pressure nadir, may be critical in determining cerebral tolerance during CPB. In that study, which included 31 patients undergoing open heart surgery, abnormal EEG patterns—including disappearance of fast waves, slowing to under 6 Hz with high voltage, or flattening—were observed in 64% of cases within the first five minutes of CPB initiation. While the lowest MAP values did not differ significantly between patients with and without EEG abnormalities (36.2 vs. 38.9 mmHg), the rate of MAP decline was significantly faster in those who developed EEG changes (0.66 mmHg/s vs. 0.34 mmHg/s, *p* < 0.01). This finding suggests that rapid circulatory changes at the onset of CPB may disrupt cerebral autoregulation, leading to EEG abnormalities even when absolute MAP values remain above conventionally accepted thresholds [54].

Taken together, these studies suggest that fixed MAP targets (e.g., 50–60 mmHg or 60–70 mmHg) are insufficient to ensure adequate cerebral perfusion during CPB. EEG parameters (BIS, SEF) and cerebral oxygenation metrics (rSO_2_, MCAV) provide complementary information about cerebral metabolic status and perfusion adequacy. Furthermore, the rate of MAP change—not just its absolute value—emerges as an important determinant of cerebral well-being. Personalized strategies that integrate real-time monitoring of cerebral autoregulation, EEG patterns, oxygenation variability, and hemodynamic stability may be more effective than rigid pressure thresholds in preventing neurological injury. The MAP and cerebral perfusion in CPB are summarized in Table 3.

### 4.3. Hemodilution and Hematocrit Levels: Implications for Cerebral Oxygen Delivery and EEG Changes

The initial hemodilution that occurs as soon as cardiopulmonary bypass commences is unavoidable since the extracorporeal circuit is primed with a balanced electrolyte solution [55]. Mild hemodilution may be advantageous, in that it will lower blood viscosity and, consequently, increase cerebral flow [56].

A prospective study of 12 patients undergoing mitral valve surgery established the hemoglobin safety threshold for preventing EEG slowing. The study showed that during CPB, hemoglobin levels decreased significantly from a preoperative value of 11.95 ± 1.44 mg/dL to 9.39 ± 1.03 mg/dL 30 min after CPB initiation (*p* = 0.0001), and further declined to 9.13 ± 0.80 mg/dL by the end of CPB (*p* = 0.0001). Receiver operating characteristic (ROC) curve analysis identified the hemoglobin thresholds for preventing EEG slowing as 9.4 mg/dL (hematocrit 28.2%) at 30 min after CPB initiation (sensitivity 75%, specificity 75%) and 9.2 mg/dL (hematocrit 27.6%) at the end of CPB (sensitivity 71.4%, specificity 100%). This study was the first to propose a lower safety limit for hemodilution based on EEG monitoring [12].

Subsequent research shifted focus to the stability of cerebral oxygenation. A study of 71 patients undergoing cardiac valve surgery found that increased variability in regional cerebral oxygen saturation (rSO_2_) during the rewarming phase was an independent risk factor for delayed postoperative neurocognitive recovery (OR = 4.93, 95% CI: 1.25–19.42, *p* = 0.023). In contrast, neither MAP variability nor BIS variability showed significant associations with cognitive outcomes. This finding highlights that maintaining stable cerebral oxygenation during rewarming—a period of increased metabolic demand—is more critical for neuroprotection than maintaining stable blood pressure [57].

More recent research directly quantified the association between cerebral desaturation (defined as a ≥10% decrease from baseline) and EEG burst suppression, finding that cerebral desaturation increased the risk of burst suppression by 1.5-fold (OR = 1.52, 95% CI: 1.11–2.07, *p* = 0.009). Notably, the risk of concurrent cerebral desaturation and burst suppression during CPB was 52.3 times higher than before CPB (OR = 52.3, 95% CI: 19.5–140, *p* < 0.001), particularly during the period between aortic cross-clamp removal and the end of CPB (OR = 10, 95% CI: 4.01–25.1, *p* < 0.001) [58].

Taken together, these studies demonstrate that in the setting of hemodilution during CPB, maintaining hemoglobin levels at no less than 9.4 mg/dL represents a safety threshold for preventing EEG slowing; cerebral desaturation (≥10%) is significantly associated with an increased risk of burst suppression; and stable cerebral oxygenation during the rewarming phase is critical for postoperative cognitive function. These findings collectively suggest that relying solely on BIS or blood pressure targets may be insufficient to ensure cerebral protection. Individualized strategies integrating cerebral oxygenation monitoring, hemoglobin management, and hemodynamic stability may be more effective in preventing postoperative neurocognitive injury. The hemodilution and hematocrit levels in CPB are summarized in Table 4.

**Figure 2:**
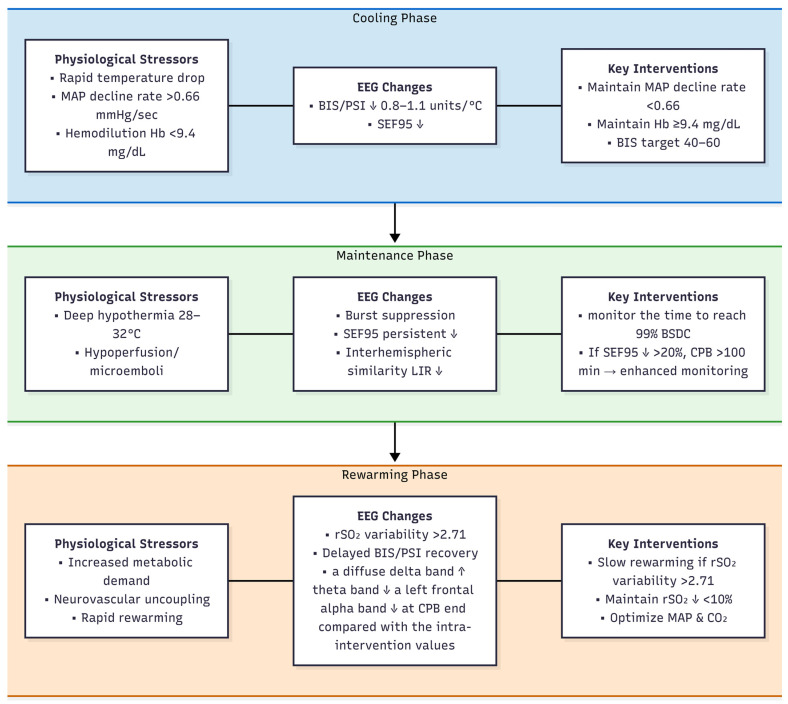
Relationships between physiological stressors, EEG changes, and clinical outcomes during CPB. (↑ indicates increase; ↓ indicates decrease; → indicates leads to).

Relationships between physiological stressors, EEG changes, and clinical outcomes during CPB are shown in Figure 2.

## 5. Technical and Pharmacological Factors Influencing EEG Indicators During CPB

### 5.1. Anesthetic Agents: Specific EEG Signatures and Their Association with Postoperative Neurological Outcomes

Although anesthetics appear to protect brain cells largely by suppressing brain activity, also called electrophysiological activity, there are important and major differences among anesthetic agents as to their mechanisms of action, their effects on CBF and metabolism, and their interaction with body temperature.

In research on the effects of anesthetic agents on the electroencephalogram, barbiturates (such as thiopental) were among the first to be systematically studied. Thiopental produces dose-dependent EEG suppression, with burst suppression occurring at high doses; when combined with hypothermia, its EEG suppressant effect is significantly enhanced, extending the duration of suppression from 1.3 min with thiopental alone or 4.8 min with hypothermia alone to 26–29 min [59]. Further studies demonstrated that thiopental reduces the cerebral metabolic rate for oxygen (CMRO_2_) while proportionally reducing cerebral blood flow (CBF)—i.e., maintaining flow–metabolism coupling—but delays emergence from anesthesia (6.4 h vs. 2.0 h) and increases the need for inotropic agents [60,61,62].

In contrast, the volatile anesthetics isoflurane and sevoflurane exhibit different EEG and cerebrovascular effects. Isoflurane induces burst suppression during hypothermic cardiopulmonary bypass (CPB) when arterial concentrations reach 46.5 ± 10.7 μg/mL [63]. Unlike thiopental, isoflurane reduces CMRO_2_ without proportionally reducing CBF—i.e., it uncouples flow–metabolism coupling—and impairs cerebral autoregulation [60,64]. Notably, isoflurane requirements decrease significantly after CPB (end-tidal concentration 0.36% vs. 0.46% pre-CPB, *p* = 0.031) [65], suggesting increased sensitivity to volatile anesthetics post-bypass. Moreover, a closed-loop isoflurane administration system targeting the BIS maintains target BIS more accurately than manual control (84.6% vs. 75.9% of time within target range, *p* < 0.01) [66]. At burst suppression concentrations (3.36 ± 0.03%), sevoflurane decreases cerebral blood flow velocity (CBFV) by 17% and cerebral oxygen extraction (COE) by 23%, and also exhibits uncoupling of flow–metabolism coupling and impaired autoregulation [67]. Of note, during CPB, BIS is insensitive to clinically relevant changes in sevoflurane plasma concentration; an increase of 40 μg/mL in sevoflurane concentration results in only a 3.6-unit decrease in BIS [68].

Propofol, a representative intravenous anesthetic, shows significant pharmacodynamic changes during CPB. Due to reduced plasma protein binding, the unbound fraction of propofol increases 2-fold, leading to enhanced efficacy [69]. Notably, the correlation between BSR and unbound propofol concentration (r^2^ = 0.56) is much stronger than that between BIS and unbound propofol concentration (r^2^ = 0.19), indicating that BSR better reflects deep anesthesia [70]. Propofol burst suppression reduces both CBF and CMRO_2_ while maintaining coupling [71], which may help reduce cerebral embolic load during surgery. Unlike volatile anesthetics, CPB itself does not alter propofol requirements or postoperative emergence time [72].

Opioids have relatively modest effects on the EEG. Fentanyl (30–70 μg/kg) produces high-voltage delta waves without burst suppression; sharp waves occurred in a dose-dependent manner (20% at 30 μg/kg, 80% at 70 μg/kg), but no seizure-related clinical signs were observed [73]. When compared at equipotent EEG-based concentrations, fentanyl and sufentanil (10:1 concentration ratio) provided equivalent hemodynamic control, but fentanyl was significantly less expensive (C$6.12 vs. C$17.47, *p* < 0.001) [74]. In a comparison of ketamine-midazolam versus sufentanil-based regimens, no significant difference was found in postoperative QEEG deterioration rates (61.9% vs. 66.7%, *p* = 0.500), but the use of isoflurane was associated with less QEEG deterioration (ρ = 0.46–0.50, *p* < 0.01) [75].

In summary, different anesthetic agents exhibit distinct EEG signatures and differential effects on CBF and CMRO_2_ during cardiac surgery; propofol and thiopental reduce cerebral metabolism with proportionate reductions in CBF (coupled effect), whereas isoflurane and sevoflurane reduce cerebral metabolism without proportionally reducing CBF (uncoupled effect) and impair cerebral autoregulation. These differences provide important guidance for the selection of anesthetic agents in clinical practice. The contrasting EEG effects of major anesthetic agents in CPB are summarized in Table 5.

### 5.2. Embolization and Systemic Inflammatory Response: Mechanisms of Brain Injury and Corresponding EEG Manifestations

Embolization and hypoperfusion are common and important pathophysiological mechanisms contributing to organ failure (including severe brain injury) that are associated with cardiopulmonary bypass [1]. CPB initiates an SIRS through the contact of blood with the artificial substrates of the extracorporeal circuit, leading to activation of the complement plus leukocyte and endothelial cells [76]. In consequence of this intense inflammatory response, multi-organ dysfunction frequently develops, with neurocognitive decline being an important component [55].

The evolution of research on EEG manifestations of embolization and inflammation during cardiac surgery spans five decades, from early case observations to modern quantitative indices. Researchers first established that EEG seizures during CPB could arise from two distinct mechanisms: acute cerebral ischemia (hypotension or emboli) with onset < 5 min, and toxic exposure from oxygenator components with onset > 2.5 h. In this study, 64% of seizures were associated with membrane oxygenators, providing a foundational framework for understanding intraoperative brain injury [77]. These findings were translated into clinical practice, demonstrating that the implementation of continuous EEG, pressure, and pO_2_ monitoring, along with refined CPB techniques, reduced stroke incidence from 1.1% (18/1688) to 0.6% (12/2011) over a 6-year period. Notably, among the 213 monitored isolated CABG patients, EEG monitoring helped identify hypotension-related ischemia: one patient exhibited marked cortical slowing (mean pressure 34 mmHg) that reversed with an elevation of MAP, enabling timely intervention [78]. A critical distinction between delirium and cognitive disorders was made, revealing different risk factors and EEG correlates. Preoperative QEEG showed that patients with cognitive disorders had significantly slower occipital peak frequency (8.8 vs. 9.2 Hz, *p* = 0.035) and higher amplitude (7.1 vs. 4.3 dB, *p* = 0.007). Intraoperative QEEG showed that patients with delirium had higher amplitude during rewarming (right side 59.8 vs. 53.0 μV, *p* = 0.035) and slower frequency at the end of CPB (2.9 vs. 3.8 Hz, *p* = 0.011). Risk factor analysis identified age ≥ 70 years (OR = 3.5, 95% CI: 1.4–8.4), female gender (OR = 2.5, 95% CI: 1.3–4.9), hemoglobin <5 mmol/L (OR = 2.6, 95% CI: 1.1–6.1), and EEG code 2/3 (OR = 3.5, 95% CI: 1.6–7.3) as independent risk factors for delirium [79]. This distinction helps explain controversies in earlier studies where neuropsychologic complications were grouped together.

More recent studies have refined predictive capabilities. Burst suppression duty cycle (BSDC) during rewarming after deep hypothermic circulatory arrest was introduced, achieving 15/16 (93.8%) accuracy in predicting postoperative delirium, with an area under the ROC curve of 0.849, demonstrating that EEG recovery dynamics are highly informative [80]. The overall incidence of epileptiform discharges was found to be 26% (16/62), with a significantly higher incidence in the delirium group (52.6%) compared to the non-delirium group (14.0%, *p* < 0.001). Multivariate regression analysis identified epileptiform discharges as an independent risk factor for delirium (OR = 5.00, 95% CI: 1.34–18.74, *p* = 0.017), highlighting the role of abnormal neuronal excitability [81].

A novel interhemispheric similarity index (the lateral interconnection ratio, LIR) was introduced, revealing distinct temporal patterns: stroke patients exhibited a decrease in LIR immediately after CPB (median decrease 0.08, interquartile range (IQR): 0.01–0.36), while delirium patients showed a decrease at the end of surgery (median decrease 0.14, IQR: 0.01–0.29). The AUC for predicting stroke was 0.771 (95% CI: 0.659–0.884, *p* < 0.0001), and for delirium it was 0.779 (95% CI: 0.663–0.895, *p* < 0.001). Combining LIR decrease with CPB duration ≥100 min increased stroke risk prediction to 15% (vs. 1–2% in other combinations), demonstrating the value of multimodal integration [82]. Further quantification showed that cardiac surgery patients had a nine-fold higher risk of delirium than non-cardiac surgery patients (41.7% vs. 4.5%, *p* = 0.0046), with delirious patients showing lower aEEG upper limits (12.16 ± 1.32 vs. 16.66 ± 1.53 μV, *p* = 0.0464), higher delta power (80.29 ± 1.62% vs. 71.02 ± 3.34%, *p* = 0.0417), and lower SEF95 (7.58 ± 0.81 vs. 10.81 ± 1.03 Hz, *p* = 0.0337), which are parameters that may serve as objective biomarkers [83].

The largest cohort evidence (n = 1161) demonstrated that 11.3% (131/1161) of patients had intraoperative EEG changes, of whom 42.7% (56/131) developed POD. The EEG demonstrated high specificity of 91.5% and a negative predictive value of 78.7%. Multivariable regression analysis showed that any EEG change nearly doubled the risk of delirium (OR = 1.97, 95% CI: 1.30–2.99, *p* = 0.001), while persistent EEG changes increased the risk by 2.65-fold (95% CI: 1.43–4.92, *p* = 0.002), further supporting its utility as a diagnostic tool for identifying high-risk patients [84]. The embolization and systemic inflammatory response in CPB are summarized in Table 6.

Additionally, although the correlation between inflammatory markers and POD or postoperative cognitive dysfunction (POCD) did not reach statistical significance in the overall cohort, patients who developed POD or POCD had significantly higher levels of interleukin-6 (IL-6) and neutrophil-to-lymphocyte ratio (NLR) at 48 h postoperatively compared to those who did not (IL-6: 196.94 vs. 160.79 pg/mL, *p* = 0.013; NLR: 14.98 vs. 8.81, *p* = 0.013), suggesting that these markers may help identify high-risk patients. Regarding clinical risk factors, patients with POD or POCD were older (66 vs. 60 years, *p* = 0.004), and had longer CPB duration (135.3 vs. 107.7 min, *p* < 0.001), longer mechanical ventilation time (27 vs. 14.9 h, *p* = 0.004), longer vasopressor support duration (26.6 vs. 17.1 h, *p* < 0.001), a higher rate of blood transfusion (81% vs. 45.5%, *p* < 0.001), and significantly elevated preoperative and postoperative creatinine levels (both *p* < 0.001). In terms of the temporal distribution of cognitive outcomes, 14 patients (15.9%) developed POD at 48 h postoperatively, 22 patients (25%) met the diagnostic criteria for POCD at 96 h postoperatively, and among these 22 patients with early POCD, 9 (40.9%) still had cognitive impairment at 3 months postoperatively, indicating that cognitive dysfunction may persist into the intermediate postoperative period in some patients. The longer-term trajectories require further investigation [85].

## 6. Discussion

### 6.1. Controversies and Unresolved Questions

The debate regarding the utility of the Bispectral Index under hypothermic and CPB conditions stems largely from the effects of temperature on EEG signals and monitoring algorithms. Studies have confirmed that BIS decreases linearly with temperature (approximately 1.12 units per 1 °C decrease, *p* < 0.001) [38], and another demonstrated a strong linear correlation between BIS and suppression ratio when the latter exceeded 50% during deep hypothermic circulatory arrest (r = –0.987) [47]. Conversely, one study found that BIS exhibited wide variability during hypothermia, with a correlation coefficient of only 0.033 with temperature (*p* > 0.05), while the auditory evoked potential index remained more stable [46]. Another report showed that at 30 min after CPB initiation, BIS values were significantly lower in the moderate hypothermia group than in the mild hypothermia group (22.4 ± 7.1 vs. 36.9 ± 11.1, *p* = 0.0007), suggesting that hypothermia may affect the BIS algorithm [50]. A further study demonstrated that during CPB, BIS is insensitive to clinically relevant changes in sevoflurane concentration (a 40 μg/mL increase resulted in only a 3.6-unit decrease), while a 1 °C increase in temperature was associated with a 0.5-unit increase in BIS [68]. Thus, under hypothermic conditions, BIS should be interpreted in conjunction with raw EEG or spectral edge frequency, and response entropy should be prioritized for monitoring noxious stimulation.

The controversy surrounding optimal mean arterial pressure during cardiopulmonary bypass centers on whether a fixed target value is sufficient to ensure individualized cerebral perfusion. One study supports maintaining MAP within the range of 50–100 mmHg as safe, with EEG changes primarily driven by temperature rather than pressure [48]. However, another study found that the rate of MAP decline (≥0.66 mmHg/s) is a stronger predictor of EEG abnormalities than the absolute pressure value, with the abnormal EEG group showing a significantly faster decline rate (0.66 vs. 0.34 mmHg/s, *p* < 0.01), while the lowest MAP values did not differ significantly between groups [54]. A further study revealed that under fixed pump flow conditions, there was no significant difference in median MAP between the delirium and non-delirium groups (74 vs. 68 mmHg, *p* = 0.22), but the delirium group exhibited significantly higher middle cerebral artery blood flow velocity and significantly lower Bispectral Index values, suggesting that even when MAP is within conventional ranges, a coexistence of cerebral overperfusion and metabolic suppression may occur [53]. Therefore, rather than pursuing a single MAP target, individualized assessment incorporating cerebral oximetry, EEG, and transcranial Doppler is recommended.

Notably, there still remain conflicting findings and unanswered questions across anesthetic regimens. First, what is the reliability of BIS, currently the most widely used indicator for monitoring anesthetic depth? Studies have shown that BIS correlates weakly with unbound propofol concentration (r^2^ = 0.19), whereas BSR exhibits a moderate correlation (r^2^ = 0.56) [69]. During deep propofol anesthesia, BIS may enter a plateau phase and lose sensitivity to changes in drug concentration, while BSR continues to reflect variations in anesthetic depth. A similar phenomenon has been observed with volatile anesthetics; during sevoflurane anesthesia, BIS is insensitive to changes in plasma concentration [68], further supporting the view that BIS has limitations in deep anesthesia states. Second, the effects of hypothermia on EEG-derived indices remain controversial; some investigators report a linear relationship between temperature and BIS [38,39] while others find no significant correlation or wide interpatient variability [46,50], suggesting that temperature correction algorithms may not be universally applicable across patient populations. Third, while propofol is consistently reported to maintain flow–metabolism coupling [69,71], one study found that propofol requirements during CPB were unchanged compared to off-pump surgery [72], whereas another reported enhanced efficacy due to increased unbound fraction [69]. These discrepancies may be attributable to differences in study design, patient populations, anesthetic regimens, and the specific EEG parameters examined. Collectively, these conflicting findings underscore the need for standardized methodologies and individualized approaches when interpreting EEG-derived indices during cardiac surgery, rather than relying on universal thresholds or algorithms.

The inconsistency regarding the correlation between EEG suppression and postoperative cognitive dysfunction primarily arises from differences in the persistence of suppression, outcome definitions, and confounding factors. One study found that monitoring the time to reach 99% burst suppression duty cycle correctly predicted delirium in 15 out of 16 cases (93.8%), demonstrating a significant association [80]. Another large cohort study confirmed that persistent EEG changes increased the risk of delirium by 2.65-fold (OR = 2.65, 95% CI: 1.43–4.92, *p* = 0.002) [84], while another reported that during CPB, the combination of cerebral desaturation (≥10% decrease) and burst suppression was associated with a 52.3-fold increased risk [58]. A further study distinguished between delirium and cognitive disorders; delirium was associated with intraoperative EEG changes (increased amplitude during rewarming and slower frequency at the end of CPB), whereas cognitive disorders were associated with preoperative EEG slowing [79].

### 6.2. Clinical Implications

**Figure 3:**
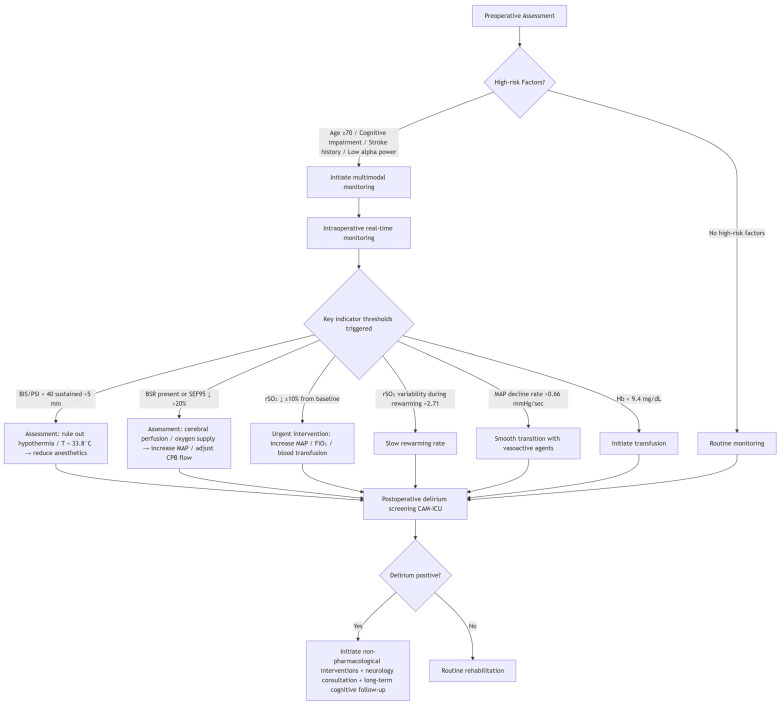
Multimodal neuromonitoring protocol for CPB management. (↓ indicates decrease; → indicates leads to).

Based on current evidence, the multimodal neuromonitoring protocol for CPB management are below (Figure 3).

The implementation of multimodal neuromonitoring in cardiac surgery should begin with preoperative risk assessment, forming a closed-loop management system that spans the entire perioperative period. Concurrently, a history of cognitive impairment and vascular risk factors such as stroke should be evaluated, as cognitive impairment increases the risk of delirium by 4.17-fold, while a history of stroke increases the risk by 2.55-fold [85]. These high-risk patients require intensified intraoperative monitoring and early postoperative intervention.

Intraoperative monitoring integrates multiple signals, including EEG, cerebral oxygenation, hemodynamics, and hemodilution. In terms of EEG monitoring, when BIS or PSI remains consistently below 40 without corresponding temperature changes, the anesthetic should be reduced after ruling out hypothermia [39,40]. By monitoring the time to reach 99% BSDC, the risk of delirium can be predicted early in the rewarming phase, providing an opportunity for intervention [80]. Even when BIS is stable, a persistent decline in SEF95 of more than 20% indicates cerebral functional suppression, warranting evaluation for hypothermia, hypoperfusion, or excessively deep anesthesia [46]. An immediate postoperative decrease in interhemispheric similarity (LIR) exceeding 0.08, combined with a CPB duration greater than 100 min, increases stroke risk to 15%, prompting urgent assessment [82]. Regarding cerebral oxygenation monitoring, a decrease in rSO_2_ of 10% or more from baseline requires immediate action: checking probe placement, increasing MAP (target > 65 mmHg), increasing FiO_2_, or considering blood transfusion [58]. rSO_2_ variability exceeding 2.71 during rewarming suggests neurovascular uncoupling, necessitating slower rewarming and optimization of blood pressure and carbon dioxide [57]. For hemodynamic monitoring, when the rate of MAP decline exceeds 0.66 mmHg/s, even if the absolute MAP remains above 50 mmHg, vasoactive agents should be used for smooth transition [54]; Absolute MAP should be maintained between 50 and 90 mmHg, with comprehensive assessment combining cerebral oxygenation and EEG findings [48]. Regarding hemodilution, when hemoglobin falls below 9.4 mg/dL, a transfusion strategy should be initiated to prevent EEG slowing [12].

The choice of anesthetic agents should be individualized based on patient risk and surgical characteristics. Propofol is preferred for high-risk patients and those requiring maintained flow-metabolism coupling, as its burst suppression ratio (BSR) reflects deep anesthesia more accurately than BIS, and its efficacy is enhanced during CPB due to increased unbound fraction [69,71]. Sevoflurane or isoflurane may be selected for patients requiring rapid awakening or mild hypothermia, but it should be noted that high concentrations of volatile anesthetics impair autoregulation and have reduced requirements after CPB [64,67]. For routine coronary artery bypass grafting, fentanyl is preferred, as its cost at equipotent EEG-based concentrations is only one-third that of sufentanil [74]. For deep hypothermic circulatory arrest, a single bolus of thiopental 15 mg/kg is sufficient without continuous infusion, though delayed emergence should be anticipated [59].

Postoperative monitoring and follow-up should extend beyond hospital discharge. Delirium screening should be performed twice daily, with particular attention to patients who experienced intraoperative epileptiform discharges, as their risk of delirium increases fivefold [81]. Postoperative quantitative EEG showing persistent slow waves or decreased alpha power indicates a risk of long-term cognitive impairment [79]. Although long-term follow-up data are limited, cognitive function assessment at 6 to 12 months is recommended for patients who experienced delirium to monitor their long-term neurocognitive trajectories [85]. This multimodal strategy, by integrating monitoring parameters with clear intervention thresholds, is expected to achieve closed-loop management from risk identification to intraoperative intervention, thereby reducing the incidence of postoperative neurological complications.

## 7. Conclusions

Cardiac surgery with CPB is a highly complex procedure which inherently has risks of cerebral injury, resulting in a spectrum of neurological complications. Using electroencephalography as a marker, with its qualitative and quantitative measures, we can then monitor this process effectively in real time, with an understanding of the “physiological state” of the brain as it adapts to this difficult environment imposed by the CPB circuit.

Analogous interactions obtain for key physiological variables such as temperature of CPB, maintenance of mean arterial pressure, and degree of hemodilution, all of which reflect their effects on cerebral metabolism, perfusion and oxygenation, and are thus pursued in EEG signatures. Nonetheless, technical features of CPB, such as choice of anesthetic agents, also leave reproducible EEG signatures and contribute to overall risk of brain injury. Finally, fundamental CPB pathophysiological processes such as embolization and SIRS (the net phenomena of which are known to account for organ dysfunction), are also reflected in characteristic EEG patterns.

Despite exciting developments, challenges remain in this area. Future work might benefit from focusing on well-designed interventional studies that optimize CPB management based on real-time neurophysiological data. Such studies are critical if the aim of ultimately improving patient safety and preserving neurological function after cardiac surgery is to be realized.

## Figures and Tables

**Table 1:** Key EEG parameters and their clinical significance in CPB.

Parameter	Typical Interpretation/Significance	Relevance in CPB	Clinical Strategy
EEG Frequency Bands			
Delta Waves	Deep sleep, motivational processes, unconstrained urges	Prospective observational study: Delta band diffuse ↑ at CPB end (T2 vs. T1, *p* < 0.05); also ↑ vs. pre-CPB (*p* < 0.05); reflects neuronal metabolic suppression [12].	Monitor the degree of delta wave slowing. An increase greater than 10% of slow EEG frequency suggests neuronal impairment; promptly assess for cerebral oxygen supply deficiency.
Theta Waves	Memory, emotional regulation, salience detection	Prospective observational study: Theta band diffuse ↓ at CPB end (T2 vs. T1, *p* < 0.05); mild anterior ↑ at 30 min post-CPB onset (ns.) [12].	Pay attention to theta power reduction at the end of CPB. A significant decrease warrants vigilance for postoperative cognitive decline.
Alpha Waves	Resting state, inversely related to cortical activation, attentional control	Retrospective cohort study: Preoperative alpha power ↓ predicts CPB burst suppression, per 1 dB increase: 12% risk ↓ (OR = 0.88, 95% CI: 0.79–0.98) and 11% duration ↓ (IRR = 0.89, 95% CI: 0.84–0.93); Prospective observational study: A left frontal alpha band ↓ were observed after CPB end (T2 vs. T1, *p* < 0.05) [16].	For patients with low preoperative alpha power, avoid excessively deep anesthesia intraoperatively and actively implement cerebral protection strategies to reduce the risk of burst suppression and delirium.
Beta Waves	Sensorimotor behavior, alertness, attentional activation	Retrospective cohort study: Preoperative beta power ↓ associated with burst suppression susceptibility—reflects impaired cortical pyramidal/interneuron integrity [16].	Similar to alpha waves. Low preoperative beta power indicates neural vulnerability; maintain cortical excitability during surgery.
Bispectral Index (BIS)	Single dimensionless number (0–100) integrating multiple EEG characteristics	Clinical applications (multiple studies): Closed-loop system: functional 96.1%; BIS within target ± 10 for 86% of time (excluding the CPB period) [19]. BIS info ↓ event frequency by 30% (5.00→3.49/patient, *p* < 0.001); propofol consumption ↓ in BIS-visible group (*p* < 0.0167) [20]. Late CPB: BIS unchanged (36.0 vs. 35.1, *p* = 0.695) but SEF95 ↓ 14.6 → 10.6 Hz (*p* = 0.0022)—BIS fails to reflect EEG slowing 62% good agreement, 33% non-agreement, 5% disagreement [21]. SNAP™ II 28% higher than BIS (95% CI 24–33%); r = 0.61 [23]. BIS more affected by frontal EMG (r^2^ = 0.62 vs. entropy r^2^ = 0.39–0.45); monitoring failure: 9.1% (BIS) vs. 0.1% (entropy) of anesthesia time (*p* < 0.0001) [24]. BIS vs. entropy: good correlation (r^2^ = 0.66–0.70) but wide LoA (BIS-SE: +16/−11.8; BIS-RE: +14.3/−14.2); RE most sensitive to noxious stimulation (skin incision d = −0.71, sternotomy d = −0.94, *p* < 0.001) [25].	Combine with raw EEG or SEF95 during hypothermia/late CPB; Interpret with caution when neuromuscular blockade is insufficient; Prioritize use with response entropy (RE) to monitor noxious stimulation.
Quantitative EEG Parameters			
Burst Suppression Ratio (BSR)	Quantifies periods of high activity (bursts) alternating with inactivity (suppression)	Retrospective cohort study: preoperative alpha and beta power (OR = 0.88, 95% CI: 0.79–0.98; IRR = 0.89, 95% CI: 0.84–0.93) predicted a later incidence and longer duration of burst-suppression. Other clinical variables—such as gender, depression, and diabetes—showed group differences [16].	Patients with low preoperative alpha/beta power are at high risk. Avoid deep anesthesia during surgery and maintain CPB temperature ≥ 33.8 °C.
Spectral Edge Frequency (SEF)	Frequency below which 90% or 95% of total EEG power lies	Prospective observational study: Late CPB SEF95 ↓ from 14.6 → 10.6 Hz (*p* = 0.0022)—SEF more sensitive than BIS for detecting EEG slowing, serves as supplementary indicator for CPB brain monitoring [21].	When BIS is stable but SEF95 continuously declines, rule out hypothermia and cerebral hypoperfusion; adjust anesthetics or vasoactive medications accordingly.

↑ indicates increase; ↓ indicates decrease; → indicates a change to.

**Table 2:** Summary of temperature management in CPB.

Study (Year)	Study Design	Patient Population	Primary Endpoint	Key Effect Size and Confidence Level	Clinical Implication
Kileny et al. (1983) [45]	Prospective observational	12 cardiac surgery patients	To evaluate the use of MLR during hypothermic surgery	Correlation coefficient between Pa latency and temperature: r = −0.617 (*p* < 0.01)	MLR can monitor cerebral perfusion status during mild-to-moderate hypothermic surgery
Markand et al. (1984) [44]	Prospective observational	16 cardiac surgery patients	To evaluate multimodal evoked potentials during hypothermia	SSEP: recordable at 20–25 °C vs. disappeared <20 °C	BAEP and short-latency SSEP are more reliable for monitoring brain function during hypothermia
Levy (1984) [49]	Prospective observational	33 cardiac surgery patients	To quantify EEG power spectrum changes during hypothermia	Total power: 1215 μV^2^ per 1 °C ↑ (*p* < 0.0001); High-frequency peak frequency: 0.39 Hz per 1 °C ↑ (*p* < 0.002)	Hypothermia-induced EEG changes can be distinguished from acute ischemic events
Russ et al. (1987) [48]	Prospective observational	39 CABG patients	To analyze correlation between SEF and body temperature	Cooling phase: r = 0.76 (*p* = 0.26 × 10−^9^); Rewarming phase: non-linear (*p* = 0.011)	SEF can be used to monitor brain function changes during hypothermia
Mizrahi et al. (1989) [41]	Prospective observational	56 aortic surgery patients	To determine peripheral temperature range at ECS onset	Esophageal temperature range at ECS: 7.2–23.1 °C (mean 13.6 °C)	EEG is a reliable guide for determining safe level of hypothermia during deep hypothermic circulatory arrest
Markand et al. (1990) [42]	Prospective observational	14 cardiac surgery patients	To quantify relationship between SSEP latency and temperature	N20 latency: 1.56 ms per 1 °C ↓ (*p* < 0.0001)	Provides quantitative basis for temperature correction of SSEP
Doi et al. (1997) [46]	Prospective observational	12 cardiac surgery patients	To compare 4 depth-of-anesthesia monitors during CPB/hypothermia	BIS-temperature correlation: r = 0.033 (ns.)	AEPIndex may be a more reliable depth-of-anesthesia monitor during hypothermia
Schmidlin et al. (2001) [39]	Prospective observational	28 CABG patients	To compare BIS differences between hypothermic and normothermic CPB	Median BIS: 41 (hypothermic) vs. 49 (normothermic) (*p* < 0.0001)	Hypothermia significantly affects BIS; anesthetic depth should be adjusted accordingly
Mathew et al. (2001) [38]	Prospective observational	100 cardiac surgery patients	To evaluate effect of temperature on BIS	BIS: 1.12 units per 1 °C ↓ (*p* < 0.001)	Quantifies the independent effect of hypothermia on BIS
Honan et al. (2006) [50]	Prospective observational	30 CABG patients	To compare effect of mild vs. moderate hypothermia on BIS	BIS at 30 min CPB: moderate hypothermia (28–30 °C) 22.4 ± 7.1 vs. mild hypothermia (32–34 °C) 36.9 ± 11.1 (*p* = 0.0007); Before X-clamp release: 19.0 ± 11.7 vs. 39.5 ± 17.4 (*p* < 0.0001); After X-clamp release: 14.4 ± 6.5 vs. 43.5 ± 13.0 (*p* = 0.0007); Overall group difference: estimate 7.85 (*p* = 0.0015)	BIS values during hypothermia should be interpreted with caution; temperature effects on the algorithm should be considered
Hayashida et al. (2007) [47]	Prospective observational	20 aortic surgery patients	To evaluate effect of DHCA on BIS and SR	SR ≥ 50%: r = −0.987 (BIS = 50.8 − 0.51 × SR); SR < 50%: r = −0.460	BIS can track suppression and recovery of cerebral electrical activity during DHCA
Zanatta et al. (2014) [43]	Retrospective observational	84 cardiac surgery patients	To evaluate effect of steady-state hypothermia on SSEP	N20 amplitude: hypothermia (32 °C) 3.2 ± 1.6 μV vs. normothermia (36 °C) 2.5 ± 1.4 μV (+26%) (*p* < 0.001); N20 latency: 25.9 ± 2.5 ms vs. 22.0 ± 1.8 ms (+17%) (*p* < 0.001)	Increased SSEP amplitude during steady-state hypothermia should not be misinterpreted as neurological improvement
Belletti et al. (2023) [40]	Prospective observational	28 elective cardiac surgery patients	To quantify effect of temperature changes on SedLine parameters	PSI: 0.84 points per 1 °C ↓ during cooling (*p* < 0.001); SR: 2.9% per 1 °C ↓ during cooling (*p* < 0.001)	Clinicians should consider temperature effects when interpreting PSI and SR values
Qin et al. (2024) [51]	Retrospective cohort	26 adult PDA surgery patients	To compare neuroprotective effects of different hypothermia strategies	rSO2-AUC: moderate hypothermia (26–31 °C) 259.04 ± 56.22 vs. mild hypothermia (32–35 °C) 185.33 ± 71.81 (*p* = 0.009); Delirium incidence: 6.67% vs. 36.36% (ns.)	Moderate hypothermia (26–31 °C) may offer better cerebral protection than mild hypothermia (32–35 °C) in adult PDA surgery

↑ indicates increase; ↓ indicates decrease.

**Table 3:** Summary of MAP and Cerebral Perfusion in CPB.

Study (Year)	Study Design	Patient Population	Primary Endpoint	Key Effect Size and Confidence Level	Clinical Implication
Russ et al. (1987) [48]	Prospective observational	39 CABG patients	To analyze correlation between SEF and perfusion pressure (PP) during hypothermic CPB	MAP maintained at 50–100 mmHg; PP maintained >40 mmHg during CPB; SEF and PP: no correlation during cooling or rewarming	EEG (SEF) is independent of PP across a wide range (50–100 mmHg), allowing differentiation of hypothermic EEG slowing from acute ischemic changes
Suzuki et al. (1991) [54]	Prospective observational	31 open heart surgery patients (aged 5–20 years)	To analyze relationship between hemodynamic changes at CPB onset and EEG abnormalities	MAP decrease rate: abnormal EEG group 0.66 mmHg/s vs. normal EEG group 0.34 mmHg/s (*p* < 0.01); Incidence of EEG abnormalities: 64% (20/31) within first 5 min of CPB; Lowest MAP: abnormal EEG group 36.2 mmHg vs. normal EEG group 38.9 mmHg (ns.); CVP change rate: abnormal EEG group 2.8 vs. 0.16 cmH_2_O/s (ns.)	Rapid decline in MAP (≥0.66 mmHg/s) at CPB onset is a stronger predictor of EEG abnormalities than the absolute MAP nadir; rapid circulatory changes may disrupt cerebral autoregulation
Thudium et al. (2024) [53]	Prospective observational	36 cardiac surgery patients (13 with POD, 23 without)	To assess association between cerebral perfusion (TCD, NIRS, BIS) and postoperative delirium	MAP: no significant difference between POD (74 mmHg) and non-POD (68 mmHg) (*p* = 0.22); CPB pump flow fixed at 2.5 L/min/m^2^ in both groups; MCAV significantly higher in POD (10.655 cm/s, 95% CI: 0.491–20.819); BIS significantly lower in POD (−4.449, 95% CI: −7.978 to −0.925)	Despite similar MAP and fixed pump flow, POD patients exhibited cerebral overperfusion (higher MCAV) with reduced cortical metabolism (lower BIS), suggesting mismatch between fixed flow and individual metabolic demand

**Table 4:** Summary of hemodilution and hematocrit levels in CPB.

Study (Year)	Study Design	Patient Population	Primary Endpoint	Key Effect Size and Confidence Level	Clinical Implication
Del Felice et al. (2016) [12]	Prospective comparative	12 elective mitral valve surgery patients	To determine minimum hemoglobin level to avoid EEG slowing	Hb levels: T0 11.95 ± 1.44 mg/dL; T1 9.39 ± 1.03 mg/dL (*p* = 0.0001); T2 9.13 ± 0.80 mg/dL (*p* = 0.0001); ROC cutoff T1: Hb 9.4 mg/dL (Ht 28.2%), sensitivity 75%, specificity 75% (AUC = 0.7188); ROC cutoff T2: Hb 9.2 mg/dL (Ht 27.6%), sensitivity 71.4%, specificity 100% (AUC = 0.7857)	Maintaining Hb ≥9.4 mg/dL (Ht ≥28%) during CPB and ≥9.2 mg/dL (Ht ≥27.6%) at CPB end prevents EEG slowing indicative of neuronal dysfunction
Zhang et al. (2021) [57]	Prospective observational	71 cardiac valve surgery patients	To evaluate association between rSO_2_ variability and delayed postoperative neurocognitive recovery	Baseline rSO_2_: PNCD 72.37 ± 7.07% vs. non-PNCD 72.09 ± 6.28% (*p* = 0.863); rSO_2_ variability (rewarming): PNCD 2.71 vs. non-PNCD 1.68 (*p* = 0.030); Multivariate OR: high rSO_2_ variability (OR = 4.93, 95% CI: 1.25–19.42); MAP variability: no significant difference across phases (*p* = 0.111–0.491)	Greater rSO_2_ variability during rewarming is an independent risk factor for delayed neurocognitive recovery (OR = 4.93); stable cerebral oxygenation is more critical than MAP or BIS stability
Ramachandran et al. (2025) [58]	Retrospective analysis	51 cardiac surgery patients	To investigate association between cerebral desaturation (≥10% decrease) and burst suppression	Desaturation vs. burst suppression: (OR = 1.52, 95% CI: 1.11–2.07); CPB vs. pre-CPB desaturation (OR = 22.1, 95% CI: 12.4–39.2); Concurrent desaturation + burst suppression (CPB) (OR = 52.3, 95% CI: 19.5–140); Post-cross-clamp period: desaturation (OR = 6.59, 95% CI: 3.62–12); Inhalational agent (per 0.1% ↑): burst suppression (OR = 7.81, 95% CI: 6.26–9.74)	Cerebral desaturation (≥10% drop) is strongly associated with burst suppression, especially during CPB and post-cross-clamp period; targeted interventions to prevent desaturation may reduce burst suppression and improve cognitive outcomes

↑ indicates increase.

**Table 5:** Contrasting EEG effects of major anesthetic agents in CPB.

Agent	Key EEG Signature	Effect on CBF/CMRO_2_	Effect on Cerebral Autoregulation	Clinical Implication
Propofol	Burst suppression at high doses [70]; BIS correlates weakly with unbound concentration (r^2^ = 0.19), BSR correlates strongly (r^2^ = 0.56) [69]	Normothermia: CBF decreased by 43%, CMRO_2_ decreased by 44%, C(a-v)O_2_ unchanged, indicating maintained flow-metabolism coupling; Hypothermia: CBF and CMRO_2_ further decreased, with coupling maintained; Rewarming: CBF and CMRO_2_ recovered but remained below control levels, with coupling maintained [71]	Preserved: C(a-v)O_2_ and SjvO_2_ unchanged vs. control; coupling maintained [71]	Propofol reduces CBF and CMRO_2_ with maintained coupling [71]; BSR is more sensitive than BIS for deep anesthesia [69];CPB itself does not alter propofol requirements [72]
Sevoflurane	Burst suppression at high concentrations (3.36 ± 0.03% for burst suppression) [67]; BIS insensitive to SPC changes [68]	Uncoupled: CBFV ↓ 17% (*p* < 0.05); COE ↓ 23% (*p* < 0.05); CBF exceeds metabolic demand [67]	Impaired: Autoregulation slope more positive with sevoflurane (0.26 ± 0.04 vs. 0.09 ± 0.03 cm/s/mmHg, *p* < 0.01) [67]	Sevoflurane impairs cerebral autoregulation and causes loss of flow-metabolism coupling [67] BIS poorly reflects sevoflurane concentration during CPB; temperature significantly affects BIS [68]
Isoflurane	Burst suppression at arterial concentration 46.5 ± 10.7 μg/mL [63]; onset 27.3 ± 4.56 min; elimination t½ 18.8 ± 5.46 min [59]	Hypothermia: CBF decreased by 27%, COE decreased by 13%, CBF still exceeds metabolic demand [64]; Normothermia: Despite reduced CMRO_2_, CBF similar to control, indicating uncoupling of flow-metabolism coupling [60]	Impaired: Autoregulation slope more positive with isoflurane (0.25 ± 0.04 vs. 0.19 ± 0.04 cm/s/mmHg, *p* < 0.05) [64]	Isoflurane reduces CMRO_2_ without proportionally reducing CBF (uncoupling), impairs autoregulation [60,64]; requirement decreases after CPB [65]; closed-loop administration improves BIS target accuracy [66]
Thiopental	Burst suppression at 8 mg/kg; profound suppression with hypothermia: 26.1–29.3 min vs. 1.3 min alone [59]	Coupled: CBF ↓ 57% (8.2 ± 2.5 vs. 14.6 ± 5.5 mL/100g/min, *p* < 0.05); CMRO_2_ ↓ 34% (0.27 ± 0.02 vs. 0.41 ± 0.08 mL/100g/min) [60]; C(a-v)O_2_ increased (3.9 ± 2.0 vs. 2.6 ± 1.1 mL/dL, *p* = 0.032) [61]	Preserved: CBF reduction proportionate to CMRO_2_ reduction; C(a-v)O_2_ widening indicates increased oxygen extraction [61]	Thiopental reduces both CBF and CMRO_2_ with maintained coupling [60,61]; combined with hypothermia produces prolonged EEG suppression [59]; emergence time delayed [61]; single bolus (15 mg/kg) before aortic declamping provides equivalent protection to continuous infusion, with faster extubation [62]
Fentanyl	High-voltage slow delta waves; sharp waves dose-related (20% at 30 μg/kg, 80% at 70 μg/kg); no burst suppression [73]	Minimal direct effect on CBF; maintains hemodynamic stability; requires isoflurane supplementation for hypnosis [74]	Preserved; does not impair autoregulation [74]	Fentanyl produces EEG depression without burst suppression; provides unconsciousness and amnesia; no intraoperative awareness reported [73]; more cost-effective than sufentanil at equipotent EEG-based doses [74]
Sufentanil	Similar to fentanyl: high-voltage slow waves; EEG effects dose-dependent [74]	Minimal direct effect on CBF; maintains hemodynamic stability [74]	Preserved [74]	Sufentanil provides equivalent hemodynamic control to fentanyl at 10:1 concentration ratio; significantly more expensive; no advantage over fentanyl in routine CABG surgery [74]
Ketamine + Midazolam	QEEG deterioration comparable to sufentanil-based anesthesia [75]	Unknown; isoflurane use correlated with less QEEG deterioration [75]	Unknown	No difference in QEEG marker of neurologic injury between ketamine-midazolam and sufentanil-based anesthesia; isoflurane use associated with better QEEG outcomes [75]

↓ indicates increase.

**Table 6:** Summary of embolization and systemic inflammatory response in CPB.

Study (Year)	Study Design	Patient Population	Primary Endpoint	Key Effect Size and Confidence Level	Clinical Implication
Stockard et al. (1974) [77]	Prospective observational	280 cardiac surgery patients (11 with EEG seizures)	To investigate epileptiform EEG activity during CPB	Ischemic seizures: 4 patients; Toxic seizures: 7 patients; Seizure onset during CPB: <5 min (ischemic) vs. >2.5 h (toxic); Association with membrane oxygenator: 9/11 seizures; Diazepam: reduced seizure activity	EEG seizures during CPB may result from acute cerebral ischemia (hypotension/emboli) or toxic substances from oxygenator components; EEG monitoring can identify seizure activity masked by neuromuscular blockade
Okies et al. (1986) [78]	Prospective cohort	3699 cardiac surgery patients (1979–1985; 30 strokes)	To assess impact of EEG, pressure, and pO_2_ monitoring on stroke risk	Stroke incidence: 1979–1981 1.1% (18/1688), 1982–1985 0.6% (12/2011); EEG monitoring (1982–1985): closed group: 213 monitored, 156 not; open group: 457 monitored; EEG changes during CPB: hypotension-related slowing resolved with MAP elevation; Probable causes identified in 7/12 strokes, including 5/5 monitored patients	Implementation of continuous EEG, arterial/venous oximetry, and pO_2_ monitoring, along with refined CPB techniques, reduced stroke incidence from 1.1% to 0.6%; EEG monitoring helps identify hypotension-related ischemia and embolic events, enabling timely intervention
Hofsté et al. (1997) [79]	Prospective observational	321 cardiac surgery patients (44 delirium, 68 cognitive disorders)	To examine pre- and intraoperative QEEG as predictors of delirium and cognitive disorders	Delirium risk factors: age ≥ 70 (OR = 3.5, 95% CI: 1.4–8.4), female gender (OR = 2.5, 95% CI: 1.3–4.9), Hb < 5 mmol/L (OR = 2.6, 95% CI: 1.1–6.1), EEG code 2/3 (OR = 3.5, 95% CI: 1.6–7.3); Cognitive disorder risk factors: age ≥ 70 (OR = 3.6, 95% CI: 1.8–7.5), CPB ≥ 2.5 h (OR = 3.7, 95% CI: 1.5–9.5), pH <7.35 (OR = 2.9, 95% CI: 1.2–6.8); Preoperative QEEG: occipital peak frequency slower (8.8 vs. 9.2 Hz, *p* = 0.035), higher amplitude (7.1 vs. 4.3 dB, *p* = 0.007) in cognitive disorder group; Intraoperative QEEG (delirium): higher amplitude during rewarming (right 59.8 vs. 53.0 μV, *p* = 0.035), slower frequency at end CPB (2.9 vs. 3.8 Hz, *p* = 0.011)	Delirium and cognitive disorders have different risk factors; preoperative QEEG predicts cognitive disorders (occipital slowing); intraoperative QEEG predicts delirium (higher amplitude, slower frequency); Hb < 5 mmol/L is a treatable risk factor for delirium
Ma et al. (2020) [80]	Retrospective analysis	16 DHCA patients (5 delirium positive, 11 negative)	To predict POD using burst suppression duty cycle (BSDC) during rewarming	T_1_ (time to 99% BSDC): delirium negative mean 73.6 ± 31.4 min; Prediction accuracy: 15/16 cases correct using BSDC milestones; AUC: 0.849 (γ = 1), AUH: 0.889; Accuracy by γ: γ = 1/4: 0.85, γ = 1/2: 0.89, γ = 3/4: 0.89	Burst suppression dynamics during rewarming predict POD; time to reach BSDC milestones correlates with delirium; prolonged transition from ECS to full BSDC indicates higher risk
Li et al. (2022) [81]	Prospective observational	62 cardiac surgery patients (19 delirium, 43 no delirium)	To investigate association between intraoperative epileptiform discharges and postoperative delirium	Epileptiform discharge incidence: 26% (16/62); Delirium incidence: 31% (19/62); Epileptiform discharges in delirium group: 52.63% vs. non-delirium 13.95% (*p* < 0.001); Univariate (OR = 6.85, 95% CI: 1.97–23.84), *p* = 0.002; Multivariate (OR = 5.00, 95% CI: 1.34–18.74), *p* = 0.017; Age (OR = 4.75, 95% CI: 1.26–17.92), *p* = 0.022	Epileptiform discharges during cardiac surgery are independently associated with postoperative delirium; EEG monitoring may identify patients at increased risk for POD
Baron Shahaf et al. (2023) [82]	Retrospective observational	803 cardiac surgery patients (31 stroke, 48 delirium)	To evaluate LIR as predictor of stroke and delirium	Stroke: LIR decrease start to post-CPB 0.08 (0.01, 0.36) vs. no-dysfunction −0.04 (−0.13, 0.04); Delirium: LIR decrease start to end 0.14 (0.01, 0.29) vs. −0.02 (−0.11, 0.08); AUC stroke: 0.771 (95% CI: 0.659–0.884), *p* < 0.0001; AUC delirium: 0.779 (95% CI: 0.663–0.895), *p* < 0.001; LIR drop + CPB ≥100 min: stroke risk 15% (vs. 1–2% in other combinations)	Prefrontal EEG LIR decreases after CPB in patients who develop stroke, and at surgery end in those who develop delirium; timing of LIR decrease may differentiate injury mechanisms; combination with CPB duration improves risk prediction
Xue et al. (2023) [83]	Prospective observational	46 postoperative ICU patients (24 cardiac, 22 non-cardiac; 11 delirium)	To identify qEEG parameters predicting POD	Cardiac vs. non-cardiac POD incidence: 41.7% vs. 4.5% (*p* = 0.0046); Delirium vs. non-delirium: aEEG upper limit 12.16 ± 1.32 vs. 16.66 ± 1.53 μV (*p* = 0.0464); delta power 80.29 ± 1.62% vs. 71.02 ± 3.34% (*p* = 0.0417); SEF95 7.58 ± 0.81 vs. 10.81 ± 1.03 Hz (*p* = 0.0337); RAV: 24.92 ± 2.43% (cardiac) vs. 33.38 ± 2.77% (non-cardiac, *p* = 0.0295)	Cardiac surgery with CPB carries higher delirium risk; qEEG parameters (aEEG upper limit, delta power, SEF95) correlate with POD; RAV reduction may reflect cerebral blood flow changes; qEEG may enable early POD detection
Al-Qudah et al. (2024) [84]	Retrospective cohort	1161 cardiovascular surgery patients (275 POD, 886 no POD)	To determine utility of intraoperative EEG in predicting POD	EEG changes: 11.28% (131/1161); POD with EEG changes: 42.74% (56/131); POD without EEG changes: 21.26% (219/1030); Sensitivity: 20.4% (95% CI: 15.9–25.4%); Specificity: 91.5% (95% CI: 89.6–93.2%); NPV: 78.7%; Adjusted OR (any EEG change): 1.97 (95% CI: 1.30–2.99), *p* = 0.001; Adjusted OR (persistent EEG change): 2.65 (95% CI: 1.43–4.92), *p* = 0.002	Intraoperative EEG changes, especially persistent changes, double the risk of POD; high specificity (91.5%) suggests EEG is valuable for identifying high-risk patients; transient changes may be reversed by timely intervention

## Data Availability

No new data were created or analyzed in this study.

## Abbreviations

The following abbreviations are used in this manuscript:

CPB	Cardiopulmonary bypass
CNS	Central nervous system
CABG	Coronary artery bypass grafting
SIRS	Systemic inflammatory response syndrome
EEG	Electroencephalography
PRISMA	Preferred Reporting Items for Systematic Reviews and Meta-Analyses
BIS	Bispectral Index
SE	state entropy
RE	response entropy
qEEG	Quantitative EEG
BSR	Burst Suppression Ratio
OR	odds ratio
CI	confidence interval
IRR	incidence ratio
SEF	Spectral Edge Frequency
PSI	patient state index
SSEP	Somatosensory evoked potential
BAEP	Brainstem auditory evoked potentials
MLR	middle-latency auditory evoked response
SR	suppression ratio
ECS	Electrocerebral silence
MAP	Mean arterial pressure
ROC	Receiver operating characteristic
CBF	Cerebral blood flow
CBFV	cerebral blood flow velocity
COE	cerebral oxygen extraction
HCT	Hematocrit
CMRO_2_	Cerebral metabolic rate for oxygen
BSDC	Burst suppression duty cycle
LIR	lateral interconnection ratio
POCD	postoperative cognitive dysfunction
POD	Postoperative delirium
IQR	interquartile range
IL-6	interleukin-6
NLR	neutrophil-to-lymphocyte ratio
NS	Not significant
